# Associação entre a Mortalidade por Doenças Crônicas Não Transmissíveis e o Índice de Desenvolvimento Humano no Brasil entre 1980 e 2019

**DOI:** 10.36660/abc.20211009

**Published:** 2023-04-12

**Authors:** Sandra Chagas da Costa Feliciano, Paolo Blanco Villela, Gláucia Maria Moraes de Oliveira

**Affiliations:** 1 Universidade Federal do Rio de Janeiro Rio de Janeiro RJ Brasil Universidade Federal do Rio de Janeiro, Rio de Janeiro, RJ – Brasil

**Keywords:** Doenças Não Transmissiveis, Indicadores de Desenvolvimento, Saúde Pública, Saúde de Pessoa com Deficiência, Epidemiologia, Mortalidade

## Abstract

**Fundamento:**

No Brasil, em 2019, as doenças crônicas não transmissíveis (DCNT) acarretaram mais de 734 mil óbitos, 55% de todas as mortes, com importante impacto socioeconômico.

**Objetivos:**

Analisar as taxas de mortalidade das DCNT, no Brasil, de 1980 a 2019, e sua associação com indicadores socioeconômicos.

**Métodos:**

Trata-se de um estudo descritivo, de séries temporais dos óbitos por DCNT, no Brasil, de 1980 a 2019. Os dados relativos às frequências anuais de mortes e da população foram obtidos do DATASUS. Foram estimadas as taxas de mortalidade brutas e padronizadas por 100.000 habitantes, pelo método direto (população do Brasil de 2000). Foram calculados os quartis de cada DCNT, onde a mudança de quartil, por aumento das taxas de mortalidade, foi representada por gradiente cromático. O Índice de Desenvolvimento Humano Municipal (IDHM) de cada unidade da federação (UF) foi extraído do site Atlas Brasil e correlacionado com as taxas de mortalidade por DCNT.

**Resultados:**

Ocorreu redução nas taxas de mortalidade por doenças do aparelho circulatório no período, exceto na região Nordeste. Houve também aumento da mortalidade por neoplasia e diabetes, enquanto as taxas das doenças respiratórias apresentaram poucas variações. Houve correlação inversa entre as UF com maior redução nas taxas de mortalidade por DCNT e o IDHM.

**Conclusões:**

A redução observada na mortalidade por doenças do aparelho circulatório pode refletir melhoria dos indicadores socioeconômicos, no Brasil, nesse período. O aumento da taxa de mortalidade por neoplasias provavelmente se relaciona com o envelhecimento da população. As maiores taxas de mortalidade por diabetes parecem ser associadas com o aumento da prevalência da obesidade nas mulheres brasileiras.

## Introdução

As doenças crônicas não transmissíveis (DCNT) são representadas pelas doenças do aparelho circulatório (DAC), neoplasias (NEO), diabetes mellitus (DM) e doenças respiratórias crônicas (DRC).^1^ Em geral, caracterizam-se por longo período de latência e evolução, lesões irreversíveis e complicações que acarretam diferentes graus de incapacidade ou óbito,^2^ e são consideradas um grande problema de saúde pública, por representarem as principais causas de morte e incapacidade prematura em todo o mundo.^3^

Em 2015, foram estimados 41 milhões de óbitos por DCNT no mundo, 36,6% destes em pessoas com idade entre 30 e 69 anos, e mais de 85% dessas mortes prematuras ocorreram em países de baixa e média renda, causando impacto no desenvolvimento econômico e no sistema de saúde.^4 , 5^ No Brasil, o Departamento de Informática do Sistema Único de Saúde (DATASUS), em 2019, atribuiu às DCNT mais de 734 mil óbitos, o que representou 55% de todas as mortes ocorridas no país. As DAC, representadas principalmente pelas doenças isquêmicas do coração (DIC) e pelas doenças cerebrovasculares (DCBV), ocupam o primeiro lugar, com mais de 362 mil óbitos nesse período.^6^

São vários os fatores de risco comuns às DCNT. Além dos fatores de riscos clássicos – modificáveis ou comportamentais, existem os determinantes da saúde, que são fatores sociais, econômicos, culturais, educacionais, étnicos/raciais, psicológicos e comportamentais e influenciam de forma indireta nas condições de saúde da população, e são interdependentes para a ocorrência de doenças.^7 - 9^

Estudos realizados no Brasil demonstraram que a melhoria dos indicadores socioeconômicos se associou a redução da mortalidade por DAC,^10^ e que as taxas de mortalidade por DAC, DCBV, e doenças hipertensivas variaram de maneira inversa ao Índice de Desenvolvimento Humano (IDH).^11^ Apesar deste fato, no Brasil, existem poucos estudos que relacionaram as taxas de mortalidade por DCNT com o IDH.^10 - 12^

O conhecimento do comportamento e a distribuição das doenças na população por um longo período são úteis na proposição de políticas públicas, assim como na avaliação, gestão e planejamento de ações de promoção e prevenção dos serviços de saúde. Desta forma, este estudo tem por objetivo analisar as taxas de mortalidade das DCNT no Brasil, no período de 1980 a 2019, avaliando a relação entre as taxas de mortalidade por cada DCNT e suas associações com o IDH.

## Métodos

Trata-se de um estudo ecológico, descritivo e de séries temporais dos registros de óbitos por DCNT, em todas as faixas etárias e em ambos os sexos, ocorridos no Brasil, no período de 1980 a 2019. Os dados relativos às frequências anuais de mortes foram obtidos do Sistema de Informações sobre Mortalidade (SIM), disponibilizados no site do DATASUS do Ministério da Saúde.^6^

Para classificação dos óbitos no período de 1980 a 1995 foram utilizadas as categorias da 9ª Revisão da Classificação Estatística Internacional de Doenças e Problemas Relacionados à Saúde (CID-09): DAC (Capítulo VII), DIC (Categorias 410-414), DCBV (430-438), NEO (Categorias 140-208; 239), DM (Categoria 250) e DRC (Categorias 490-516 e 518-519), (OMS, 1978). Para os óbitos no período de 1996 a 2019 foram utilizadas as categorias da 10ª. Revisão da Classificação Estatística Internacional de Doenças e Problemas Relacionados à Saúde (CID-10): DAC (Categorias 100-199), DIC (Categorias I20 a I25), DCBV (Categorias I60-I69), NEO (Categorias C00-C97), DM (Categorias E10-E14) e DRC (Categorias J30-J98), sendo desconsiderados os óbitos com o sexo ignorado em todo o recorte temporal. As taxas de mortalidade por todas as causas, também foram obtidas para o mesmo período do estudo.^13 , 14^

Os dados referentes à população do Brasil, das regiões geográficas (RG) – Norte, Nordeste, Sudeste, Sul, e Centro-Oeste – e unidades federativas (UF), foram obtidos do DATASUS, baseados nos censos de 1980, 1991, 2000 e 2010, projeções intercensitárias até 2012, e projeções populacionais de 2013 em diante, do Instituto Brasileiro de Geografia e Estatística (IBGE). Os dados foram estratificados por sexo em oito grupos etários (até 19 anos; 20 a 29 anos; 30 a 39 anos; 40 a 49 anos; 50 a 59 anos; 60 a 69 anos; 70 a 79 anos; e 80 anos ou mais).

Foram estimadas as taxas anuais brutas e padronizadas de mortalidade por 100.000 habitantes das DCNT (DAC, NEO, DRC e DM), no decorrer dos 40 anos de observação, por RG e UF pelo método direto, em ambos os sexos, utilizando a população do Brasil do ano 2000 como padrão. Cabe ressaltar que, para comparação das taxas de mortalidade em diferentes populações é indispensável a padronização por uma população padrão com os mesmos grupos etários, a fim de anular o efeito da distribuição demográfica desigual. Assim, o produto final é o número de óbitos esperados caso as taxas brutas encontradas para cada região geográfica fossem aplicadas à mesma população (no caso, a do ano 2000).^15^

Observou-se a relação entre as taxas de mortalidade por DCNT padronizadas por 100.000 habitantes, entre 1980, 1994 e 2019 e sua associação com o total de óbitos no Brasil. Os dados de Tocantins foram considerados somente após o ano de 1989.

Para cada UF do Brasil nos anos de 1980, 1994 e 2019, foram calculadas as taxas de mortalidade padronizadas por 100.000 habitantes para cada DCNT, que foram representadas por cores diferentes. Para permitir uma comparação proporcional entre as DCNT, foram calculadas as medianas e os quartis para DAC, DM, NEO e DRC, e a variação entre os quartis ao longo dos anos foi representada pela variação na intensidade das respectivas cores, deixando-as mais intensas em caso de aumento das taxas com mudança de quartil, e menos intensas em caso de redução nas taxas com consequente mudança de quartil.

Para cada região foram estimadas as taxas brutas de mortalidade nos sexos masculino e feminino, de acordo com as DCNT, em oito períodos de cinco anos, de 1980 a 2019, com posterior cálculo da razão das taxas. Ressalta-se que a partir de 1989, a região Norte passou a computar os dados de Tocantins, UF criada em 1988.

Por fim, foram construídos gráficos de dispersão e calculados os coeficientes de correlação de Pearson para cada DCNT e o IDH de 2010, adotando nível de significância estatística de 5%. O IDH e suas dimensões estadual e municipal, também denominadas IDHM, foram extraídos do site Atlas Brasil.^16 , 17^ Assim, foram avaliadas as relações entre as taxas de mortalidade por cada DCNT e o Índice de Desenvolvimento Humano Municipal (IDHM) de cada UF. Para a análise dos dados e construção das tabelas e gráficos, foi utilizado o programa Microsoft Office Excel® 2016 (Microsoft Corporation, EUA).

## Resultados

**Figura 1:**
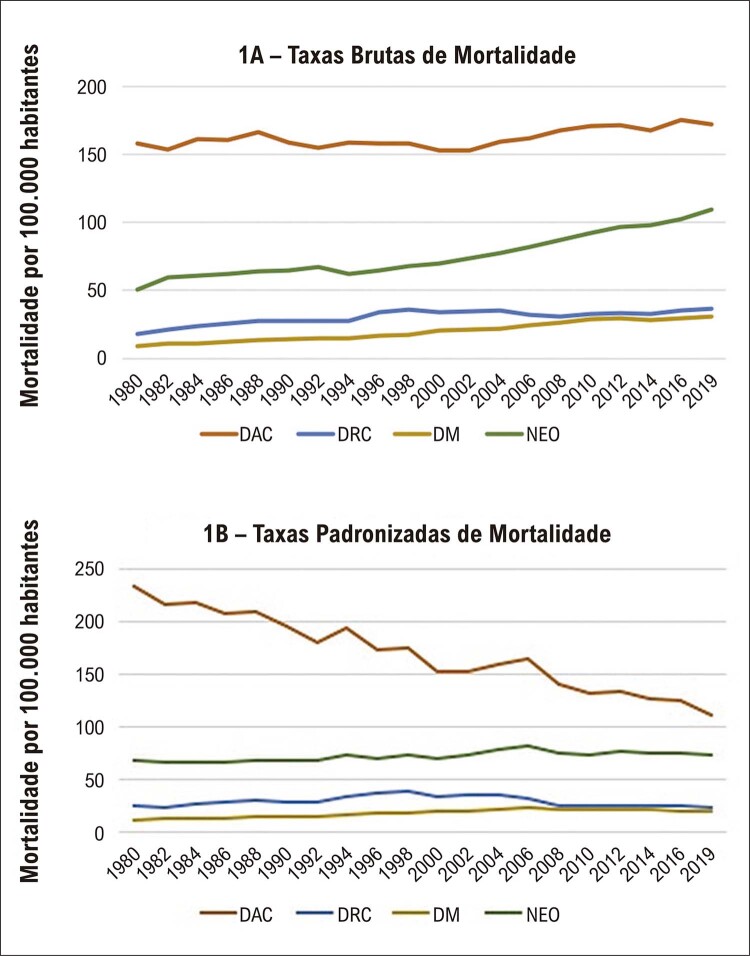
– Taxas brutas (1A) e padronizadas (1B) de mortalidade por 100,000 habitantes das doenças crônicas não transmissíveis (DAC: doenças do aparelho circulatório; DRC: doenças respiratórias crônicas; DM: diabetes mellitus e NEO: neoplasias), em ambos os sexos, em todas as faixas etárias, no Brasil, no período de 1980 a 2019.

As taxas brutas de todas as DCNT, no decorrer dos 40 anos de observação, apresentaram leves oscilações, mas com tendência a elevação, sobretudo para as DAC e as NEO. Com a padronização das taxas de mortalidade é observada queda nas DAC enquanto as demais apresentam leves oscilações com tendência a estabilidade, de uma forma geral, no período estudado ( Figura 1 ).

Observou-se declínio na taxa de mortalidade padronizada das DCNT com 339,27 por 100.000 habitantes no ano de 1980 para 229,35 por 100.000 habitantes em 2019, fato que também ocorreu com as taxas padronizadas por demais causas, de 425,47 por 100.000 habitantes em 1980 para 222,08 mortes por 100.000 habitantes em 2019. Nota-se que as DAC apresentaram redução das taxas, e as DRC que mostraram oscilações com elevação em 1994 e queda em 2019. As DAC apresentaram redução de 67% para 49% nos anos observados, enquanto as NEO apresentaram elevação de 21 % para 31% na mortalidade proporcional ( Figura Central e Material Suplementar – Figuras 1A e 1B ).

**Figura Central:**
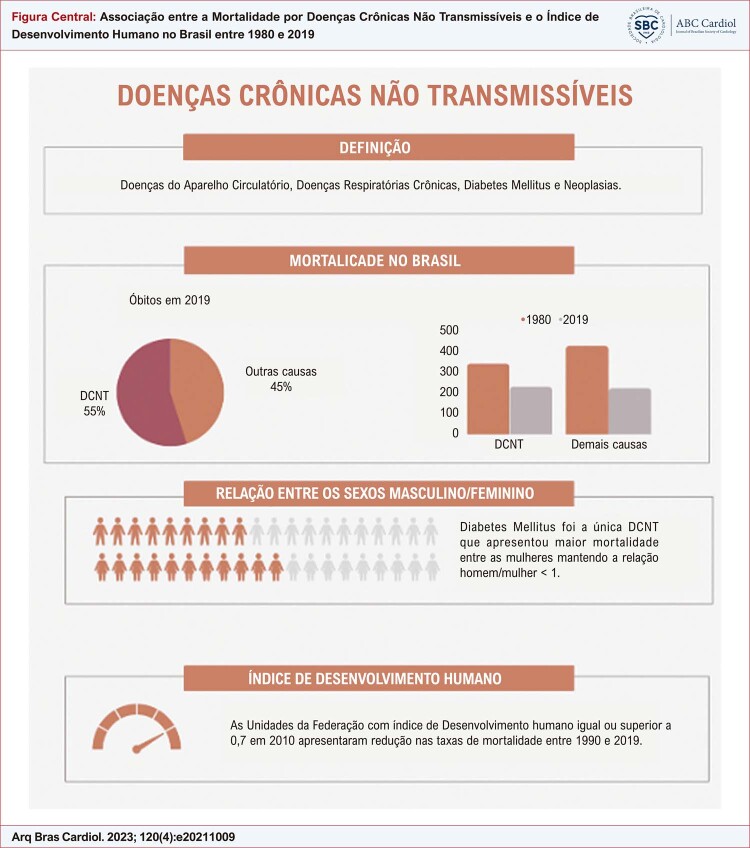
: Associação entre a Mortalidade por Doenças Crônicas Não Transmissíveis e o Índice de Desenvolvimento Humano no Brasil entre 1980 e 2019

Definição das doenças crônicas não transmissíveis; mortalidade no Brasil com percentual de óbitos em 2019 e taxas de mortalidade padronizadas por idade em 1980 e 2019; relação entre os sexos masculino/feminino das taxas de mortalidade; e possível associação da mortalidade com o Índice de Desenvolvimento Humano.

Na Tabela 1 , observa-se que a região Norte apresentou aumento dos quartis das taxas de mortalidade por NEO e DM, em todas as UF. Rondônia, Acre e Amapá apresentaram aumento das taxas por DRC, sobretudo no último ano, e ao observar as taxas de mortalidade por DAC, nota-se queda em todas UF no último ano, exceto em Roraima e Tocantins ( Tabela 1 ).

**Tabela 1:** – Taxas padronizadas de mortalidade por 100.000 habitantes, por UF, distribuídas de acordo com as doenças crônicas não transmissíveis (cores) e seus respectivos quartis (intensidade das cores) - Brasil, 1980, 1994 e 2019.

UF / Ano	1980				1994				2019			
Rondônia	189,71	25,73	49,39	3,37	143,17	33,24	51,50	23,36	111,45	29,56	74,92	26,50
Acre	143,14	22,62	52,66	1,98	143,72	23,85	42,03	8,25	124,60	48,59	71,93	22,25
Amazonas	192,64	10,60	54,54	4,06	106,57	17,16	59,38	13,05	100,69	21,57	74,04	32,09
Roraima	156,06	25,42	50,58	2,36	142,99	18,60	63,28	14,51	159,57	27,07	97,83	34,84
Pará	144,72	10,18	41,74	5,28	125,39	17,96	46,25	13,23	116,01	23,48	60,50	27,99
Amapá	140,80	11,40	60,64	13,71	161,30	28,47	85,82	14,99	116,38	32,35	72,58	27,97
Tocantins	-	-	-	-	82,68	7,84	25,07	6,65	129,23	24,02	63,25	32,18
Maranhão	64,95	3,71	13,95	2,89	62,01	5,71	18,52	4,76	136,99	18,98	57,22	34,25
Piauí	67,44	4,56	14,76	2,15	91,90	8,75	23,76	7,35	149,33	19,54	68,84	31,53
Ceará	70,91	6,29	23,88	2,97	83,99	11,12	34,99	6,63	119,58	22,31	77,32	16,29
Rio Grande do Norte	82,29	8,62	29,75	4,05	102,18	9,90	40,97	15,69	118,35	16,79	73,45	26,88
Paraíba	81,41	4,33	22,58	5,17	80,50	8,25	23,41	8,39	126,14	21,99	72,36	28,95
Pernambuco	135,83	12,94	34,49	10,22	162,37	20,83	49,15	19,47	133,16	36,72	72,86	27,94
Alagoas	123,47	10,06	33,41	10,12	125,41	15,92	31,67	15,28	153,87	21,57	61,66	39,11
Sergipe	110,36	11,59	32,05	13,43	97,30	16,95	43,12	17,55	113,30	20,85	66,71	28,97
Bahia	111,81	13,88	31,28	5,83	115,46	18,92	36,30	13,89	99,32	21,59	61,49	22,66
Minas Gerais	256,67	29,72	69,83	11,63	206,35	37,25	68,44	15,30	94,92	22,85	68,27	16,60
Espírito Santo	219,94	24,11	60,68	5,22	206,60	22,49	74,42	17,20	109,86	18,55	75,88	19,58
Rio de Janeiro	354,95	44,29	102,68	27,00	265,42	53,50	98,24	32,09	123,65	20,58	74,46	22,18
São Paulo	328,54	24,47	94,45	19,58	246,49	38,42	97,23	20,25	114,23	21,66	76,74	14,64
Paraná	299,72	23,03	88,41	9,27	266,11	43,23	92,01	15,77	105,50	24,16	79,38	19,36
Santa Catarina	244,17	31,96	74,37	10,94	205,84	44,60	94,10	18,21	99,62	23,63	82,05	17,27
Rio Grande do Sul	294,03	41,78	109,92	6,74	232,87	55,84	118,45	15,94	93,77	26,37	89,52	20,88
Mato Grosso do Sul	241,86	28,07	66,09	8,19	224,07	36,40	79,30	16,38	127,05	24,28	75,53	18,88
Mato Grosso	132,64	15,48	30,87	2,82	159,20	24,64	46,30	12,78	109,91	27,64	68,32	23,48
Goiás	168,28	20,26	44,23	5,18	218,97	31,28	65,08	14,59	115,68	31,61	73,81	20,39
Distrito Federal	252,91	46,44	102,71	20,86	242,99	38,02	109,77	29,10	74,66	16,71	67,97	13,61
**Quartis das doenças crônicas não transmissíveis de acordo com cores e intensidade das cores**												
Doenças do aparelho circulatório	**0-24%**			**25-49%**			**50-75%**			**>75%**		
Doenças respiratórias crônicas	**0-24%**			**25-49%**			**50-75%**			**>75%**		
Neoplasias	**0-24%**			**25-49%**			**50-75%**			**>75%**		
Diabetes mellitus	**0-24%**			**25-49%**			**50-75%**			**>75%**		

Na região Nordeste, todas as DCNT apresentaram aumento nas taxas de mortalidade, com exceção para as DAC na Bahia e Pernambuco, que permaneceu no mesmo quartil nos três anos observados. DM foi a doença que atingiu os maiores quartis em todas as UF à exceção do Ceará que, apesar do aumento, não atingiu o maior quartil. Após DM, as NEO, seguidas da DRC e DAC foram, na sequência, nesta região, as doenças que mais afetaram a população. Ainda na região Nordeste, Pernambuco se destaca por ser a UF que obteve os maiores quartis em todas as doenças ( Tabela 1 ).

Por outro lado, nas regiões Sudeste, Sul e Centro-Oeste, com elevadas taxas em 1980, houve tendência a redução da mortalidade por todas as DCNT, como observado pela redução da intensidade de cores dos quartis, exceto para as NEO nas duas primeiras regiões que apresentaram estabilidade e, no Centro-Oeste da DRC em Goiás e DM em Mato Grosso. O DF se sobressai com queda em todas as DCNT ( Tabela 1 ).

A Tabela 2 mostra a razão entre os sexos masculino/feminino. Assim, os números superiores a 1,0 significam que os valores para o sexo masculino são superiores aos femininos, e os números inferiores a 1,0 que os valores no sexo feminino são maiores que no masculino.

**Tabela 2:** – Razão entre as taxas brutas de mortalidade nos sexos masculino e feminino, de acordo com as doenças crônicas não transmissíveis, em períodos de cinco anos, por região geográfica

	Região / Período	1980-1984	1985-1989	1990-1994	1995-1999	2000-2004	2005-2009	2010-2014	2015-2019
Doenças do aparelho circulatório	Norte	1,14	1,15	1,18	1,19	1,26	1,29	1,31	1,29
	Nordeste	1,12	1,16	1,17	1,11	1,12	1,11	1,13	1,13
	Sudeste	1,19	1,21	1,19	1,15	1,15	1,14	1,13	1,12
	Sul	1,22	1,19	1,16	1,12	1,11	1,09	1,08	1,10
	Centro-Oeste	1,27	1,30	1,31	1,26	1,31	1,31	1,29	1,28
Doenças respiratórias crônicas	Norte	1,24	1,25	1,26	1,22	1,28	1,31	1,28	1,20
	Nordeste	1,22	1,25	1,25	1,16	1,14	1,13	1,11	1,03
	Sudeste	1,48	1,53	1,49	1,40	1,38	1,36	1,29	1,20
	Sul	1,77	1,76	1,76	1,55	1,52	1,45	1,32	1,19
	Centro-Oeste	1,28	1,30	1,32	1,32	1,35	1,36	1,29	1,28
Neoplasias	Norte	1,12	1,10	1,06	1,06	1,11	1,09	1,11	1,09
	Nordeste	0,99	1,01	1,03	1,02	1,05	1,09	1,10	1,08
	Sudeste	1,26	1,26	1,22	1,23	1,23	1,22	1,19	1,14
	Sul	1,43	1,39	1,36	1,34	1,36	1,34	1,31	1,24
	Centro-Oeste	1,13	1,12	1,17	1,19	1,20	1,23	1,22	1,17
Diabetes mellitus	Norte	0,74	0,70	0,75	0,75	0,80	0,80	0,85	0,91
	Nordeste	0,77	0,77	0,75	0,74	0,73	0,76	0,80	0,84
	Sudeste	0,68	0,68	0,70	0,73	0,78	0,82	0,83	0,86
	Sul	0,67	0,66	0,67	0,70	0,74	0,78	0,81	0,88
	Centro-Oeste	0,79	0,72	0,72	0,76	0,79	0,84	0,88	0,95

As taxas no sexo masculino foram maiores em todo o período para as DAC, DRC e NEO. A exceção foi NEO no período de 1980 a 1984 na região Nordeste com razão inferior a 1. Para as taxas de mortalidade relacionadas a DM, em todo o período observado, foram superiores no sexo feminino em todas as regiões geográficas.

**Figura 2:**
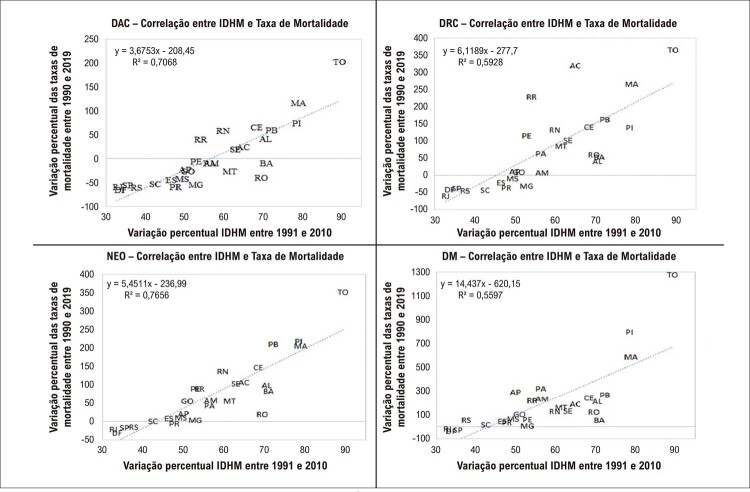
– Gráfico de dispersão. Correlação entre a variação percentual do Índice de Desenvolvimento Humano Municipal (IDHM) 1991 e 2010 com a variação percentual da taxa de mortalidade das doenças crônicas não transmissíveis: doença do aparelho circulatório (DAC); doenças respiratórias crônicas (DRC); neoplasias (NEO); diabetes mellitus (DM), 1990 e 2019, no Brasil nas UF, em ambos os sexos.

**Figura 3:**
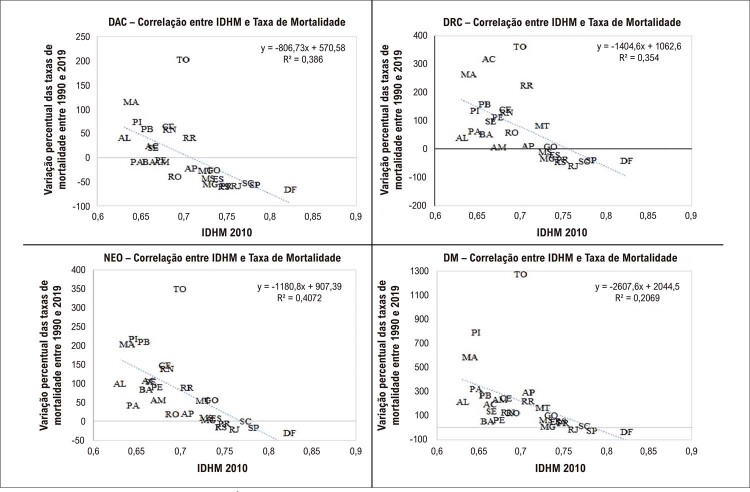
– Gráfico de dispersão. Correlação entre Índice de Desenvolvimento Humano Municipal (IDHM) absoluto no ano de 2010 com a variação percentual da taxa de mortalidade das doenças crônicas não transmissíveis: doença do aparelho circulatório (DAC); doenças respiratórias crônicas (DRC); neoplasias (NEO); diabetes mellitus (DM), 1990 e 2019, no Brasil nas UF, em ambos os sexos.

Na Figura 2 nota-se que, em todos os casos, as UF que apresentaram maior redução nas taxas de mortalidade foram as que apresentaram a menor variação no IDHM, como Rio de Janeiro, São Paulo, Distrito Federal, Rio Grande do Sul, Santa Catarina, Espírito Santo e Paraná. Ao contrário, Maranhão, Piauí e Paraíba, que apresentaram as maiores variações no IDHM, apresentaram as menores quedas nas taxas de mortalidade ( Figura 2 ). Adicionalmente, a Figura 3 demonstra redução nas taxas de mortalidade em todas as UF que apresentaram IDHM igual ou acima de 0,7, como nas regiões Sudeste, Sul e Centro-Oeste. As UF com menor variação na mortalidade eram as que tinham o maior IDHM ( Figura 3 ). Os coeficientes de correlação encontrados entre o IDHM de 2010 e as variações percentuais nas taxas de mortalidade foram: −0,62 para DAC, −0,59 para DRC, −0,45 para DM e −0,64 para NEO, todos com nível de significância inferior a 0,05.

## Discussão

O presente estudo evidenciou o declínio das taxas de mortalidade padronizadas por DCNT no Brasil, em todas as idades e sexo, no período de 40 anos de observação. Estes resultados são semelhantes ao encontrado por Cardoso et al.,^18^ no qual os autores observaram redução nas taxas de doenças cardiovasculares em todas as regiões brasileiras, entretanto, estas apresentaram as taxas médias mais altas em ambos os períodos, seguidas pelas taxas de NEO, DM e DRC.

A queda das taxas padronizadas de mortalidade por DCNT também foi observada por Malta et al.,^19 , 20^ que descreveram as tendências de mortalidade entre 1990 e 2017 no Brasil. Além da redução de 35% nas taxas de mortalidade padronizada das DCNT, os autores evidenciaram redução na mortalidade por doenças cardiovasculares em 47,9% e a mortalidade por DRC, DM e NEO diminuiu 41,3%, 18% e 11,9%, respectivamente, com 15 anos ininterruptos de queda, com tendência de aumento dos óbitos para todas as DCNT, em cada grupo, após 2015. Em nosso estudo, por outro lado, observou-se elevações nas taxas de mortalidade por NEO e DM ( Figura 1 ).

As taxas de mortalidade por DCNT foram avaliadas por Silva e Ramalho,^21^ que analisaram a situação do perfil de morbimortalidade do Brasil e compararam as modificações ocorridas para a mortalidade proporcional dos grandes grupos de causa, através da representação do crescimento da população desde 1980 com projeções até 2033. Os autores observaram que a mortalidade por DCNT já representava cerca de 70% da mortalidade total do país e que as DAC são o grupo de mortalidade com maior peso proporcional durante todo o período, com projeção de redução em 2033 (113,1 por 100.000 habitantes), quando comparada com 1980 (273,9 por 100.000 habitantes). De forma semelhante, em nosso estudo, notou-se que apesar da redução das DCNT ao longo do período, a partir de 2019 suas taxas ultrapassaram as demais causas de morte ( Material Suplementar - Figura 1A ). Por outro lado, apesar da elevada taxa de mortalidade das DCNT, as DAC apresentaram declínio em seu percentual de óbito de 67% para 49% ( Material Suplementar - Figura 1B ).

Quando são avaliadas as taxas padronizadas de mortalidade, o presente estudo apresentou resultados semelhantes a um estudo transversal que utilizou estimativas do estudo Carga Global de Doenças 2015 para o Brasil.^20^ Em ambos os estudos, observaram-se taxas mais elevadas ao longo do período, entretanto com tendências a declínio para doenças cardiovasculares e doenças respiratórias crônicas, enquanto neoplasias e diabetes apresentaram taxas estáveis, com tendência a elevação, entre 1990 e 2015 no Brasil.^20^

De uma forma geral, as comparações entre as próprias DCNT são difíceis justamente pelo fato de apresentarem diferentes ordens de grandeza dos óbitos. Assim, no presente estudo, optou-se pela construção de uma tabela dinâmica com os quartis de cada DCNT, identificando as intensidades de variações entre os intervalos ao longo dos anos, permitindo a comparação proporcional entre elas ( Tabela 1 ). Na região Nordeste, todas as DCNT apresentaram aumento nas taxas de mortalidade, sendo DM a doença que atingiu os maiores quartis. Por outro lado, os menores quartis foram observados nas regiões Sudeste, Sul e Centro Oeste, com exceção do Mato Grosso. Estes resultados são semelhantes a outro estudo já mencionado.^18^

As DAC apresentaram as maiores quedas na mortalidade, preponderando com maiores mudanças no quartis nas regiões mais ricas do país (Sudeste, Sul e Centro-Oeste) e em menor grau na região Norte, enquanto a região Nordeste apresentou aumento nas taxas de mortalidade por essas doenças. Mansur et al.,^22^ em estudo que analisou os dados das tendências da mortalidade por doenças cardiovasculares em homens e mulheres com mais de 30 anos de idade nas cinco regiões do Brasil, observaram aumento na mortalidade por estas doenças na região Nordeste com reduções nas demais regiões, sendo as mais significativas nas regiões Sul e Sudeste. Em relação às DRC, observou-se tendência a aumento para regiões orte e Nordeste e queda para as regiões Sul, Sudeste e Centro-Oeste. Quanto às NEO, as regiões Sudeste e Sul apresentaram as taxas nos quartis mais elevados. Cardoso et al.,^18^confirmaram os dados descritos acima com estudo que encontrou redução nas taxas médias de mortalidade por DRC nas regiões Centro-Oeste, Norte, Sudeste e Sul. Entretanto, contrastando com o presente estudo, encontroaram redução nas taxas medias por NEO na região Sul. Estes resultados observados, podem pelo menos em parte, estar relacionados a variações nos indicadores sociais, como já demonstrado em estudo anterior.^13^

Em relação à razão da mortalidade entre homens e mulheres, para as DAC, DRC e NEO, a mortalidade prevaleceu no sexo masculino, ao contrário do ocorrido na DM onde as taxas foram mais elevadas no sexo feminino ( Tabela 2 ), semelhante aos resultados de outros estudos.^23 , 24^

Quando foram avaliadas as relações com o IDH, observou-se que a mortalidade por DCNT no Brasil apresentou comportamento epidemiológico desigual entre as regiões, como observado nas Figuras 2 e 3 . Na primeira, observa-se que há relação inversa entre a variação do IDH e a variação das taxas de mortalidade para todas as DCNT. Entretanto, nota-se o mesmo padrão de dispersão quando são observadas as posições das UF nos gráficos, com as UF das regiões Norte e Nordeste apresentando as maiores variações no IDH, entretanto com as variações mais baixas (ou inversas) nas taxas de mortalidade. Este achado pode ser explicado pelo fato da posição inicial do IDH destas regiões, que mesmo diante de uma melhora importante, ainda continuam insuficientes para provocarem impactos positivos nas taxas de mortalidade quando observados isoladamente.

Ao contrário, quando as variações nas taxas de mortalidade são comparadas ao valor absoluto do IDH ( Figura 3 ), nota-se relação direta entre as variáveis. Nesta figura, para todas as DCNT, observa-se, de uma forma geral, que as UF que apresentaram IDH em 2010 acima de 0,7 apresentaram as melhores variações nas taxas de mortalidade, demonstrando que, provavelmente, mais importante que a variação no IDH é o valor que ele atinge, como observado em estudos anteriores.^12 , 26 - 31^

O presente estudo não aborda as causas múltiplas de óbito, não sendo possível avaliar a presença de mais de uma DCNT nas mesmas declarações de óbito. Além disso, ao trabalhar com a causa básica selecionada e disponibilizada no DATASUS, o banco de dados se torna mais sensível à incompletude das declarações de óbito e aos seus erros de preenchimento. Por fim, a indisponibilidade de dados referentes ao IDHM nos últimos anos não permite comparações temporais mais próximas com as taxas de mortalidade.

## Conclusões

A mortalidade por DCNT diminuiu ao longo dos 40 anos de observação, sobretudo nas DAC. Quanto às DRC, apresentaram leves oscilações, enquanto as NEO e DM apresentaram elevação ao longo do período. No final do período do estudo, tendem a estabilização. Os estados das regiões Nordeste e Norte tenderam a apresentar taxas de mortalidade mais elevadas quando comparados aos das regiões Sudeste, Sul e Centro Oeste. O estudo aponta correlação do IDH com a redução nas taxas de mortalidade das DCNT, sobretudo as DAC, a partir de um nível alto de desenvolvimento humano. Estes achados apontam não somente para a importância de medidas que reduzam a mortalidade por DCNT, mas que estas incluam necessariamente medidas que melhorem as condições socioeconômicas da população, considerando o contexto regional e visando reduzir as desigualdades crescentes no acesso aos recursos de prevenção e tratamento.
