# Supplementary material for: Associação entre a Mortalidade por Doenças Crônicas Não Transmissíveis e o Índice de Desenvolvimento Humano no Brasil entre 1980 e 2019
Source: Arq Bras Cardiol. 2023 Apr 12;120(4):e20211009. [Article in Portuguese] doi: 10.36660/abc.20211009 (PMC10263428; doi:10.36660/abc.20211009)
Supplement: Translation

# Translation: Association between Mortality from Chronic Noncommunicable Diseases and Human Development Index in Brazil between 1980 and 2019

**Authors:** Sandra Chagas da Costa Feliciano, Paolo Blanco Villela, Gláucia Maria Moraes de Oliveira

**Affiliations:** 1 Brazil Universidade Federal do Rio de Janeiro, Rio de Janeiro, RJ – Brazil

**Keywords:** Non Communicable Diseases, Development Indicators, Public Health, Health of the Disabled, Epidemiology, Mortality

## Abstract

**Background:**

Chronic noncommunicable diseases (CNCDs) caused more than 734,000 deaths (55% of all deaths) in Brazil in 2019, with an important socioeconomic impact.

**Objectives:**

To analyze the mortality rates from CNCDs in Brazil from 1980 to 2019 and their association with socioeconomic indicators.

**Method:**

This was a descriptive, time-series study of deaths from CNCDs in Brazil from 1980 to 2019. Data on the annual frequencies of deaths and on population were obtained from the Department of Informatics of the Brazilian Unified Health System. Crude and standardized mortality rates per 100,000 inhabitants were estimated using the direct method (Brazilian population in 2000). The quartiles of each CNCD were calculated, where a quartile change, due to an increase in mortality rate, was represented by a chromatic gradient. The Municipal Human Development Index (MHDI) of each Brazilian federative unit was extracted from the Atlas Brasil website and correlated with the rates of CNCD mortality.

**Results:**

There was a reduction in mortality rates due to diseases of the circulatory system during the period, except in the Northeast Region. There was also an increase in mortality from neoplasia and diabetes, while the rates of chronic respiratory diseases showed little variation. There was an inverse correlation between the federative units with greater reduction in CNCD mortality rates and the MHDI.

**Conclusions:**

The observed decrease in mortality due to diseases of the circulatory system may reflect an improvement in socioeconomic indicators in Brazil during the period. The increase in mortality rates due to neoplasms is probably related to the aging of the population. The higher mortality rates of diabetes seem to be associated with an increase in the prevalence of obesity in Brazilian women.

## Introduction

Chronic noncommunicable diseases (CNCDs) are represented by diseases of the circulatory system (DCS), neoplasms, diabetes mellitus (DM), and chronic respiratory diseases (CRDs).^1^They are generally characterized by a long period of latency and progression, irreversible lesions, and complications that lead to different degrees of disability or death,^2^and they are considered a major public health problem, as they represent the main causes of death and premature disability worldwide.^3^

In 2015, there were an estimated 41 million deaths from CNCDs worldwide, 36.6% of which occurred in people aged 30 to 69 years; more than 85% of these premature deaths occurred in low- and middle-income countries, causing an impact on their economic development and health care systems.^4 , 5^In Brazil, the Department of Informatics of the Brazilian Unified Health System (DATASUS, acronym in Portuguese) attributed more than 734,000 deaths to CNCDs in 2019, representing 55% of all deaths that occurred in the country. DCS, mainly represented by ischemic heart diseases (IHDs) and cerebrovascular diseases (CBVDs), occupy first place, with more than 362,000 deaths in this period.^6^

Several risk factors are common among CNCDs. In addition to the classic modifiable or behavioral risk factors, health determinants, i.e., social, economic, cultural, educational, ethnic/racial, psychological, and behavioral factors, influence indirectly the health conditions of the population and are interdependent regarding the occurrence of diseases.^7 - 9^

Studies carried out in Brazil have demonstrated that the improvement in socioeconomic indicators has been associated with a reduction in mortality from DCS,^10^and mortality rates from DCS, CBVDs, and hypertensive diseases varied inversely to the Human Development Index (HDI).^11^Despite this fact, few studies in Brazil have related the mortality rates from CNCDs to the HDI.^10 - 12^

The knowledge of the behavior and the distribution of diseases in the population over a long period are useful for the recommendation of public policies, as well as the evaluation, management, and planning of promotional and preventive actions by health care services. Thus, the aim of this study is to analyze the mortality rates of CNCDs in Brazil between 1980 to 2019, evaluating the relationship between the mortality rates for each CNCD and their associations with the HDI.

## Methods

This was as ecological, descriptive, time-series study of records of deaths from CNCDs, across all age groups and in both sexes, that occurred in Brazil in the period from 1980 to 2019. Data related to the annual frequency of deaths were obtained from the Mortality Information System (SIM), available on the website of the DATASUS of the Brazilian Ministry of Health.^6^

For the classification of deaths from 1980 to 1995, the categories of the 9th Revision of the International Statistical Classification of Diseases and Related Health Problems (ICD-9) were used: DCS (Chapter VII), IHD (Categories 410–414), CBVD (430–438), neoplasms (Categories 140–208; 239), DM (Category 250), and CRD (Categories 490–516 and 518–519), (WHO, 1978). For deaths from 1996 to 2019, the categories of the 10th Revision of the International Statistical Classification of Diseases and Related Health Problems (ICD-10) were used: DCS (Categories 100–199), IHD (Categories I20–I25), CBVD (Categories I60–I69), neoplasms (Categories C00–C97), DM (Categories E10–E14), and CRD (Categories J30–J98). Deaths throughout the time frame without known sex information were disregarded. All-cause mortality rates were also obtained for the same study period.^13 , 14^

Data referring to the Brazilian population, from the geographic regions (North, Northeast, Southeast, South, and Central-West) and the federative units were obtained from the DATASUS, based on the censuses of 1980, 1991, 2000, and 2010, intercensal projections until 2012, and population projections by the Brazilian Institute of Geography and Statistics (IBGE, acronym in Portuguese) from 2013 onwards. The data were stratified by sex into 8 age groups as follows: up to 19 years, 20 to 29 years, 30 to 39 years, 40 to 49 years, 50 to 59 years, 60 to 69 years, 70 to 79 years, and 80 years or more.

The crude and standardized annual mortality rates from the CNCDs (DCS, neoplasms, CRD, and DM) per 100,000 inhabitants were estimated over the 40 years of observation by geographic region and federative unit, by the direct method, in both sexes, using the Brazilian population of the year 2000 as the standard. It should be emphasized that, in order to compare mortality rates in different populations, standardization by a standard population with the same age groups is indispensable to nullify the effect of uneven demographic distribution. Thus, the final product is the expected number of deaths if the crude rates found for each geographic region were applied to the same population (in this case, the year 2000).^15^

The analysis included the relationship between the standardized CNCD mortality rates per 100,000 inhabitants between 1980, 1994, and 2019 and their association with the total number of deaths in Brazil. Data from the federative unit of Tocantins were considered only after the year 1989.

Standardized mortality rates per 100,000 inhabitants were calculated for each CNCD in each Brazilian federative unit for the years 1980, 1994, and 2019 and are represented by different colors. To allow a proportional comparison between CNCDs, medians and quartiles were calculated for DCS, DM, neoplasms, and CRD. The variation between quartiles over the years was represented by the variation in the intensity of the respective colors, in which they were more intense in cases of increase in rates with change in quartiles and less intense in cases of a decrease in rates with consequent changes in quartiles.

For each region, crude mortality rates were estimated for sex (male and female), according to the CNCDs, in 8 periods of 5 years, from 1980 to 2019, with subsequent calculation of the rate ratio. It is worth noting that, from 1989 onwards, the North Region began to compute data from Tocantins, a federative unit created in 1988.

Finally, scatter plots were constructed, and Pearson’s correlation coefficients were calculated for each CNCD and the 2010 HDI, adopting a statistical significance level of 5%. The HDI and its state and municipal dimensions, also called the Municipal Human Development Index (MHDI), were extracted from the Atlas Brasil website.^16 , 17^

Thus, the relationships between the mortality rates for each CNCD and the MHDI of each federative unit were evaluated. For data analysis and construction of tables and graphs, we used Microsoft Office Excel® 2016 (Microsoft Corporation, USA).

## Results

The crude rates of all CNCDs over the 40 years of observation showed slight oscillations, but a trend toward an increase, especially for DCS and neoplasms. With the standardization of the mortality rates, a decrease in DCS was observed, while the others showed slight oscillations, with a general trend toward stability during the analyzed period ( Figure 1 ).

**Figure 1:** – Crude (1A) and standardized (1B) mortality rates from chronic noncommunicable diseases (DCS: diseases of the circulatory system; CRD: chronic respiratory diseases; DM: diabetes mellitus and NEO: neoplasms) per 100,000 inhabitants, in both sexes and across all age groups from 1980 to 2019 in Brazil.

There was a decline in the standardized mortality rate for CNCDs, from 339.27 per 100,000 inhabitants in the year 1980 to 229.35 per 100,000 inhabitants in 2019, which also occurred with the standardized rates for the other causes, from 425.47 per 100,000 inhabitants in 1980 to 222.08 deaths per 100,000 inhabitants in 2019. Notably, DCS showed a reduction in rates, and CRDs showed oscillations with an increase in 1994 and a decrease in 2019. The DCS showed a reduction from 67% to 49% in the observed years, while neoplasms showed an increase from 21% to 31% in proportional mortality ( Central Illustration and Supplementary Material – Figures 1A and 1B ).

**Central Illustration:** : Association between Mortality from Chronic Noncommunicable Diseases and Human Development Index in Brazil between 1980 and 2019 Definition of chronic noncommunicable diseases; mortality in Brazil with the percentage of deaths in 2019 and age-standardized mortality rates in 1980 and 2019; relationship between male and female sex regarding mortality rates; and possible association of mortality with the Human Development Index. CNCDs: chronic noncommunicable diseases.

As seen in Table 1 , the North Region presented an increase in the quartiles of mortality rates due to neoplasms and DM across all federative units. Rondônia, Acre, and Amapá showed an increase in CRD rates, especially in the last year, while, regarding the mortality rates for DCS, a decrease was observed across all federative units in the final year, except for Roraima and Tocantins ( Table 1 ).

**Table 1:** – Standardized mortality rates per 100,000 inhabitants, by federative unit, distributed according to chronic noncommunicable diseases (colors) and their respective quartiles (color intensity) – Brazil, 1980, 1994, and 2019.

FU/Year	1980				1994				2019			
Rondônia	189.71	25.73	49.39	3.37	143.17	33.24	51.50	23.36	111.45	29.56	74.92	26.50
Acre	143.14	22.62	52.66	1.98	143.72	23.85	42.03	8.25	124.60	48.59	71.93	22.25
Amazonas	192.64	10.60	54.54	4.06	106.57	17.16	59.38	13.05	100.69	21.57	74.04	32.09
Roraima	156.06	25.42	50.58	2.36	142.99	18.60	63.28	14.51	159.57	27.07	97.83	34.84
Pará	144.72	10.18	41.74	5.28	125.39	17.96	46.25	13.23	116.01	23.48	60.50	27.99
Amapá	140.80	11.40	60.64	13.71	161.30	28.47	85.82	14.99	116.38	32.35	72.58	27.97
Tocantins	-	-	-	-	82.68	7.84	25.07	6.65	129.23	24.02	63.25	32.18
Maranhão	64.95	3.71	13.95	2.89	62.01	5.71	18.52	4.76	136.99	18.98	57.22	34.25
Piauí	67.44	4.56	14.76	2.15	91.90	8.75	23.76	7.35	149.33	19.54	68.84	31.53
Ceará	70.91	6.29	23.88	2.97	83.99	11.12	34.99	6.63	119.58	22.31	77.32	16.29
Rio Grande do Norte	82.29	8.62	29.75	4.05	102.18	9.90	40.97	15.69	118.35	16.79	73.45	26.88
Paraíba	81.41	4.33	22.58	5.17	80.50	8.25	23.41	8.39	126.14	21.99	72.36	28.95
Pernambuco	135.83	12.94	34.49	10.22	162.37	20.83	49.15	19.47	133.16	36.72	72.86	27.94
Alagoas	123.47	10.06	33.41	10.12	125.41	15.92	31.67	15.28	153.87	21.57	61.66	39.11
Sergipe	110.36	11.59	32.05	13.43	97.30	16.95	43.12	17.55	113.30	20.85	66.71	28.97
Bahia	111.81	13.88	31.28	5.83	115.46	18.92	36.30	13.89	99.32	21.59	61.49	22.66
Minas Gerais	256.67	29.72	69.83	11.63	206.35	37.25	68.44	15.30	94.92	22.85	68.27	16.60
Espírito Santo	219.94	24.11	60.68	5.22	206.60	22.49	74.42	17.20	109.86	18.55	75.88	19.58
Rio de Janeiro	354.95	44.29	102.68	27.00	265.42	53.50	98.24	32.09	123.65	20.58	74.46	22.18
São Paulo	328.54	24.47	94.45	19.58	246.49	38.42	97.23	20.25	114.23	21.66	76.74	14.64
Paraná	299.72	23.03	88.41	9.27	266.11	43.23	92.01	15.77	105.50	24.16	79.38	19.36
Santa Catarina	244.17	31.96	74.37	10.94	205.84	44.60	94.10	18.21	99.62	23.63	82.05	17.27
Rio Grande do Sul	294.03	41.78	109.92	6.74	232.87	55.84	118.45	15.94	93.77	26.37	89.52	20.88
Mato Grosso do Sul	241.86	28.07	66.09	8.19	224.07	36.40	79.30	16.38	127.05	24.28	75.53	18.88
Mato Grosso	132.64	15.48	30.87	2.82	159.20	24.64	46.30	12.78	109.91	27.64	68.32	23.48
Goiás	168.28	20.26	44.23	5.18	218.97	31.28	65.08	14.59	115.68	31.61	73.81	20.39
Distrito Federal	252.91	46.44	102.71	20.86	242.99	38.02	109.77	29.10	74.66	16.71	67.97	13.61
**Chronic noncommunicable disease quartiles according to color and color intensity**												
Diseases of the circulatory system	**0-24%**			**25-49%**			**50-75%**			**>75%**		
Chronic respiratory diseases	**0-24%**			**25-49%**			**50-75%**			**>75%**		
Neoplasms	**0-24%**			**25-49%**			**50-75%**			**>75%**		
Diabetes mellitus	**0-24%**			**25-49%**			**50-75%**			**>75%**		

In the Northeast region, all CNCDs showed an increase in mortality rates, with the exception of DCS in Bahia and Pernambuco, which remained in the same quartile in the 3 years analyzed. Notably, DM was the disease that reached the highest quartiles in all federative units, except for Ceará, which, despite an increase, did not reach the highest quartile. After DM, neoplasms, followed by CRD and DCS, were, in this sequence and region, the diseases that most affected the population. Still in the Northeast region, Pernambuco stands out as the federative unit that obtained the highest quartiles in all diseases ( Table 1 ).

On the other hand, in the Southeast, South, and Central-West Regions, with high rates in 1980, there was a trend toward a reduction in mortality from all CNCDs, as observed by the reduction in the color intensity of the quartiles, except for neoplasms in the first two regions, which were stable, and in the Central-West for CRD in Goiás and DM in Mato Grosso. Distrito Federal stands out with a reduction in all CNCDs ( Table 1 ).

Table 2 shows the sex ratios (male/female), where numbers > 1.0 represent higher values for the male than the female sex, and numbers < 1.0 represent higher values for the female sex than the male sex.

**Table 2:** – Ratio between crude mortality rates in the male and female sex for chronic noncommunicable disease in 5-year periods and by geographic region

	Region / Period	1980-1984	1985-1989	1990-1994	1995-1999	2000-2004	2005-2009	2010-2014	2015-2019
Diseases of the circulatory system	North	1.14	1.15	1.18	1.19	1.26	1.29	1.31	1.29
	Northeast	1.12	1.16	1.17	1.11	1.12	1.11	1.13	1.13
	Southeast	1.19	1.21	1.19	1.15	1.15	1.14	1.13	1.12
	South	1.22	1.19	1.16	1.12	1.11	1.09	1.08	1.10
	Central-West	1.27	1.30	1.31	1.26	1.31	1.31	1.29	1.28
Chronic respiratory diseases	North	1.24	1.25	1.26	1.22	1.28	1.31	1.28	1.20
	Northeast	1.22	1.25	1.25	1.16	1.14	1.13	1.11	1.03
	Southeast	1.48	1.53	1.49	1.40	1.38	1.36	1.29	1.20
	South	1.77	1.76	1.76	1.55	1.52	1.45	1.32	1.19
	Central-West	1.28	1.30	1.32	1.32	1.35	1.36	1.29	1.28
Neoplasms	North	1.12	1.10	1.06	1.06	1.11	1.09	1.11	1.09
	Northeast	0.99	1.01	1.03	1.02	1.05	1.09	1.10	1.08
	Southeast	1.26	1.26	1.22	1.23	1.23	1.22	1.19	1.14
	South	1.43	1.39	1.36	1.34	1.36	1.34	1.31	1.24
	Central-West	1.13	1.12	1.17	1.19	1.20	1.23	1.22	1.17
Diabetes mellitus	North	0.74	0.70	0.75	0.75	0.80	0.80	0.85	0.91
	Northeast	0.77	0.77	0.75	0.74	0.73	0.76	0.80	0.84
	Southeast	0.68	0.68	0.70	0.73	0.78	0.82	0.83	0.86
	South	0.67	0.66	0.67	0.70	0.74	0.78	0.81	0.88
	Central-West	0.79	0.72	0.72	0.76	0.79	0.84	0.88	0.95

The rates in the male sex were higher throughout the period for DCS, CRDs, and neoplasms. One exception was for neoplasms in the period from 1980 to 1984 in the Northeast Region, which had a ratio below 1. For mortality rates related to DM, throughout the observed period, they were higher in the female sex across all geographic regions.

Table 2 - Ratio between crude mortality rates in the male and female sex for chronic noncommunicable disease in 5-year periods and by geographic region.

As seen in Figure 2 , the federative units that presented the greatest reduction in mortality rates in all cases were those that presented the smallest MHDI variation, including Rio de Janeiro, São Paulo, Distrito Federal, Rio Grande do Sul, Santa Catarina, Espírito Santo, and Paraná. In contrast, Maranhão, Piauí, and Paraíba, which presented the greatest MHDI variations, had the smallest decreases in mortality rates ( Figure 2 ). Additionally, Figure 3 shows a reduction in mortality rates in all federative units with MHDI equal to or above 0.7, including the Southeast, South, and Central-West Regions. The federative units with the lowest variation in mortality were those with the highest MHDI ( Figure 3 ). The correlation coefficients found between the 2010 MHDI and the percentage variations in mortality rates were: −0.62 for DCS, −0.59 for CRDs, −0.45 for DM, and −0.64 for neoplasms, all of which had a significance level below 0.05.

**Figure 2:** – Scatter plot. Correlation between the percentage change in the 1991 and 2010 Municipal Human Development Index (MHDI) with the percentage change in mortality rate for chronic noncommunicable diseases: diseases of the circulatory system (DCS); chronic respiratory diseases (CRD); neoplasms; diabetes mellitus (DM), 1990 and 2019, in Brazil in the federative units, in both sexes.

**Figure 3:** – Scatter plot. Correlation between the absolute Municipal Human Development Index (MHDI) in the year 2010 with the percentage change in mortality rate for chronic noncommunicable diseases: diseases of the circulatory system (DCS); chronic respiratory diseases (CRD); neoplasms; diabetes mellitus (DM), 1990 and 2019, in Brazil in the federative units, in both sexes.

## Discussion

The present study showed a decline in standardized mortality rates for CNCDs in Brazil, across all ages and in both sexes, over a 40-year period of observation. These results are similar to those found by Cardoso et al.,^18^who observed a reduction in the rates of cardiovascular diseases in all Brazilian regions; however, these showed the highest average rates in both periods, followed by the rates of neoplasms, DM, and CRD.

The decline in standardized mortality rates for CNCDs has also been observed by Malta et al.,^19 , 20^who described mortality trends between 1990 and 2017 in Brazil. In addition to the 35% reduction in the standardized mortality rates for CNCDs, the authors showed a reduction in mortality from cardiovascular diseases by 47.9%, while the mortality from CRD, DM, and neoplasms decreased by 41.3%, 18%, and 11.9%, respectively, with 15 uninterrupted years of decline, followed by a trend toward an increase in deaths for all CNCDs, in each group, after 2015. In our study, on the other hand, we observed increases in mortality rates due to neoplasms and DM ( Figure 1 ).

The mortality rates for CNCDs have been evaluated by Silva and Ramalho,^21^who analyzed the profile of morbidity and mortality in Brazil and compared the changes that occurred in the proportional mortality of the major groups of causes, through the representation of population growth since 1980 with projections until 2033. The authors observed that mortality from CNCDs already represented about 70% of the total mortality in the country and that DCS comprised the mortality group with the highest proportional weight throughout the period, with a projected reduction in 2033 (113.1 per 100,000 inhabitants) when compared with 1980 (273.9 per 100,000 inhabitants). Similarly, in our study, we noted that despite the reduction in CNCDs over the period, their rates surpassed those of other causes of death after 2019 ( Supplementary Material - Figure 1A ). On the other hand, despite the high mortality rate from CNCDs, the percentage of deaths from DCS declined from 67% to 49% ( Supplementary Material - Figure 1B ).

When the standardized mortality rates were evaluated in the present study, they showed results similar to those from a cross-sectional study that used estimates from the 2015 Global Burden of Disease study for Brazil.^20^In both studies, higher rates were observed over the period, but with declining trends for cardiovascular diseases and CRD, while neoplasms and DM showed stable rates, with a tendency to increase, between 1990 and 2015 in Brazil.^20^

In general, comparisons among CNCDs are difficult, precisely because the magnitude of the deaths differs among them. Thus, in the present study, we opted to construct a dynamic table with the quartiles of each CNCD, identifying the intensities of variations between the intervals over the years and allowing the proportional comparison between them ( Table 1 ). In the Northeast Region, all CNCDs showed an increase in mortality rates, with DM reaching the highest quartiles. In contrast, the lowest quartiles were observed in the Southeast, South, and Central-West Regions, except for Mato Grosso. These results are similar to those of another previously cited study.^18^

The DCS showed the greatest decreases in mortality, with greater changes in quartiles observed in the wealthiest regions of the country (Southeast, South, and Central-West) and, to a lesser extent, the North Region, while the Northeast Region showed an increase in mortality rates due to these diseases. Mansur et al.^22^analyzed data on trends in mortality from cardiovascular diseases in men and women older than 30 years in the 5 regions of Brazil and observed that mortality from these diseases increased in the Northeast Region and decreased in the other regions, with the most significant being in the South and Southeast Regions. Regarding CRDs, there was an upward trend for the North and Northeast Regions and a downward trend for the South, Southeast, and Central-West Regions, while for neoplasms, the Southeast and South Regions had rates in the highest quartiles. The data described above were confirmed by the study by Cardoso et al.,^18^which reported a reduction in mean mortality rates from CRD in the Central-West, North, Southeast, and South Regions. However, different than the present study, theirs found a reduction in the average rates of neoplasms in the South Region. These observed results may, at least in part, be related to variations in social indicators, as demonstrated in a previous study.^13^

Regarding the mortality ratios between men and women for DCS, CRDs, and neoplasms, mortality prevailed in the male sex, in contrast to what occurred for DM, in which the rates were higher in the female sex ( Table 2 ), which is in agreement with the results of other studies.^23 , 24^

When we evaluated the associations with the HDI, we observed that the mortality from CNCDs in Brazil showed an uneven epidemiological behavior between regions, as observed in Figures 2 and 3 . In Figure 2 , an inverse relationship is observed between the HDI variation and the variation in mortality rates for all CNCDs. However, the same pattern of dispersion occurs when the positions of the federative units in the graphs are observed, with the federative units in the North and Northeast Regions presenting the greatest HDI variations but with the lowest (or inverse) variations in the mortality rates. This finding can be explained by the fact that the initial position of the HDI of these regions which, despite an important improvement, is still insufficient to cause positive impacts on mortality rates when observed alone.

On the contrary, when variations in mortality rates are compared against absolute HDI values ( Figure 3 ), a direct relationship is observed between the variables. In this figure, for all CNCDs, it is generally observed that the federative units that presented an HDI above 0.7 in 2010 had the best variations in mortality rates, demonstrating that, probably, more important than the HDI variation is the value that the HDI reaches, as observed in previous studies.^12 , 26 - 31^

The present study does not address multiple causes of death, and it is not possible to assess the presence of more than one CNCD in the same death certificates. In addition, when working with the underlying cause selected and made available in the DATASUS, the database becomes more sensitive to the incompleteness of death certificates and errors in filling them out. Finally, the unavailability of MHDI data in recent years prevents closer temporal comparisons with mortality rates.

## Conclusions

The mortality due to CNCDs decreased over the 40 years of observation, especially those of DCS. As for CRDs, they showed slight oscillations, while those for neoplasms and DM increased over the period. At the end of the study period, they tended to stabilize. The states in the Northeast and North Regions tended to have higher mortality rates when compared with those in the Southeast, South, and Central-West Regions. The study points to a correlation between HDI and the reduction in mortality rates from CNCDs, especially DCS, based on a high level of human development. These findings indicate the importance of measures that reduce CNCD mortality and the fact that these measures should, necessarily, include improvement to the socioeconomic conditions of the population, considering the regional context, and aim to reduce the growing inequalities in access to prevention and treatment resources.
